# Purkinje cell dysfunction causes disrupted sleep in ataxic mice

**DOI:** 10.1242/dmm.050379

**Published:** 2024-06-12

**Authors:** Luis E. Salazar Leon, Amanda M. Brown, Heet Kaku, Roy V. Sillitoe

**Affiliations:** ^1^ Department of Neuroscience, Baylor College of Medicine, Houston, TX 77030, USA; ^2^ Department of Pathology and Immunology, Baylor College of Medicine, Houston, TX 77030, USA; ^3^ Jan and Dan Duncan Neurological Research Institute at Texas Children's Hospital, Houston, TX 77030, USA; ^4^ Department of Pediatrics, Baylor College of Medicine, Houston, TX 77030, USA; ^5^ Development, Disease Models and Therapeutics Graduate Program, Baylor College of Medicine, Houston, TX 77030, USA

**Keywords:** Purkinje cells, Cerebellar nuclei, Ataxia, Sleep, Circadian rhythms

## Abstract

Purkinje cell dysfunction disrupts movement and causes disorders such as ataxia. Recent evidence suggests that Purkinje cell dysfunction may also alter sleep regulation. Here, we used an ataxic mouse model generated by silencing Purkinje cell neurotransmission (*L7^Cre^;Vgat^fx/fx^*) to better understand how cerebellar dysfunction impacts sleep physiology. We focused our analysis on sleep architecture and electrocorticography (ECoG) patterns based on their relevance to extracting physiological measurements during sleep. We found that circadian activity was unaltered in the mutant mice, although their sleep parameters and ECoG patterns were modified. The *L7^Cre^;Vgat^fx/fx^* mutant mice had decreased wakefulness and rapid eye movement (REM) sleep, whereas non-REM sleep was increased. The mutants had an extended latency to REM sleep, which is also observed in human patients with ataxia. Spectral analysis of ECoG signals revealed alterations in the power distribution across different frequency bands defining sleep. Therefore, Purkinje cell dysfunction may influence wakefulness and equilibrium of distinct sleep stages in ataxia. Our findings posit a connection between cerebellar dysfunction and disrupted sleep and underscore the importance of examining cerebellar circuit function in sleep disorders.

## Teaser

**Summary:** Using a genetic mouse model of ataxia, we provide insights into the role of the cerebellum in sleep regulation, highlighting its importance as an origin for sleep disruptions in motor disorders.

**Research Simplified**

Cerebellar ataxia is a movement disorder, characterised by discoordination of motor control, which can result in difficulty with tasks such as walking and speaking. However, it is also associated with severe sleep disruptions. Purkinje cells – neurons located within the cerebellum, a region at the back of the brain – are known to have abnormal activity in ataxia and increased activity during the sleep to wake transition. Understanding whether Purkinje cells play a pivotal role in the sleep disruptions seen in humans with cerebellar ataxia may help researchers develop potential therapies that address both movement and sleep difficulties in ataxia and other related conditions.

The authors previously established a laboratory mouse model in which only Purkinje cells lose their ability to influence the activity of other neurons. This precise manipulation results in mice that have common symptoms of cerebellar ataxia such as disrupted balance and lack of motor coordination. The authors found that these ataxic mice also displayed disrupted sleep timing and patterning, with a particular impact on rapid eye movement (REM) sleep. Importantly, these sleep difficulties closely resembled those experienced by humans with ataxia.

This study revealed that a single cell type, the Purkinje cells in the cerebellum region of the brain, directly modulates the quality and quantity of sleep in the mouse model. Given the strong similarities between mouse and human sleep cycles and brain structure, further investigation into cerebellar functions during different behaviours could facilitate advancement of therapeutics for cerebellar ataxia-related sleep dysfunctions in humans.

## INTRODUCTION

The cerebellum is critical for the control of different motor functions including coordination, posture, balance and learning. However, emerging evidence overwhelmingly suggests that the cerebellum also plays a crucial role in non-motor functions, including cognitive and emotional processing (Popa et al., 2014), associative learning (Larry et al., 2019) and reward expectation (Carta et al., 2019). Recent work suggests that the cerebellum also contributes to the regulation of sleep and sleep-associated processes (Salazar Leon and Sillitoe, 2022; Song and Zhu, 2021; Strick et al., 2009). Indeed, the association between cerebellar dysfunction and sleep disturbances has been corroborated in both human patients and recently in mouse models with dystonia (Salazar Leon and Sillitoe, 2023). In humans, sleep anomalies primarily manifest as disruptions in sleep timing, resulting in daytime drowsiness; increased sleep latency, denoting difficulties in initiating sleep; and parasomnia, reflecting problems with sleep maintenance (Antelmi et al., 2017; Smit et al., 2017). Importantly, these deficits disproportionately impact rapid eye movement (REM) sleep. Except for daytime drowsiness, analogous impairments are observed in mouse models of dystonia, particularly mirroring the human deficits in REM sleep timing and latency (Salazar Leon and Sillitoe, 2023). A similar association has been observed in human patients with spinocerebellar ataxia, a neurodegenerative motor disorder characterized by uncoordinated movements. Patients with ataxia present with substantial sleep disruptions, similar to those seen in patients with dystonia (Huebra et al., 2019; Pedroso et al., 2011; Shindo et al., 2019). In particular, and again similar to human patients with dystonia, sleep disruptions in ataxia tend to involve specific impairments in REM sleep length and quality. For patients with ataxia, the severity of ataxia is also a predictor of sleep impairments (Sonni et al., 2014).

Although sleep impairments in motor diseases are typically considered to be secondary symptoms, dysfunction in normal sleep behavior substantially impacts patient health. Studies in patients with cerebellar ataxia reveal that cognitive function and depression are equally and positively correlated with sleep quality (Sonni et al., 2014). Disrupted sleep also has known associations with impairments in motor learning and motor function, including eyeblink conditioning (in mice) and finger-tapping tasks (in humans), as well as motor symptoms in patients with motor diseases (De Zeeuw and Canto, 2020; Walker et al., 2003). It is postulated that this association results from the modulation of synaptic activity during sleep (Tononi and Cirelli, 2014). Thus, it stands to reason that sleep may have a key role in mediating motor function in the context of motor diseases such as cerebellar ataxia. However, although the cerebellum has many known links to abnormal motor function, the cerebellar circuit components involved in sleep regulation remain unclear. This problem is magnified in the context of ataxia, in which cerebellar function is directly affected.

The role of Purkinje cells, the exclusive output neurons of the cerebellar cortex, is of significant interest to ataxia and sleep. These cells are central to the motor phenotypes of ataxia in both animal models (White et al., 2014) and humans (Xia et al., 2013). Although ataxia can be neurodegenerative, work in different models has revealed that disease onset and progression can be attributed to functional deficits in Purkinje cell neural signaling (Hoxha et al., 2018; White et al., 2014). Furthermore, Purkinje cell signals exhibit sleep-dependent activity, which intensifies during non-REM (NREM) sleep and at the sleep-wake transition (Mano, 1970; Zhang et al., 2020). Although there is a growing consensus on the existence of a link between cerebellar dysfunction and sleep disruption, the direct impact of Purkinje cell dysfunction on sleep regulation is yet to be elucidated. Our prior studies confirmed that olivocerebellar signaling disruptions in mice lead to both motor and sleep abnormalities (Salazar Leon and Sillitoe, 2023). However, the extent to which cerebellar circuits influence sleep remains incomplete, as the previously studied genetic modification tested the impact of manipulating a population of cerebellar inputs rather than that of a principal cell type within the cerebellum. Given the pivotal role of Purkinje cells as the primary cerebellar input processors and the sole output cells from the cerebellar cortex, we aimed to investigate their potential as a key factor in driving sleep impairments. We hypothesized that cerebellar Purkinje cell dysfunction may induce sleep disturbances across various disease states.

To investigate the relationship between Purkinje cell activity and sleep, we used a constitutively active Cre-loxP system to block inhibitory synaptic transmission of Purkinje cells. As all Purkinje cells are inhibitory neurons and represent the sole output of the cerebellar cortex, this genetic manipulation effectively eliminates communication between the cerebellar cortex and the downstream cerebellar nuclei by blocking fast neurotransmission. *Vgat* (also known as *Slc32a1*), encoding the vesicular γ-aminobutyric acid (GABA) transporter (VGAT), was deleted using a *Pcp2* (*L7*) gene-regulatory element to spatially drive and restrict Cre expression to Purkinje cells. The resulting mice had the genotype *L7^Cre^;Vgat^fx/fx^* and presented with severe ataxic motor symptoms, including widened gait, incoordination and a lack of balance. This mouse model of ataxia was previously devised by our laboratory and its ataxic motor behaviors and Purkinje cell-specific deficits have been explored in detail, including with the use of Purkinje cell-specific antibodies (Stay et al., 2019; White et al., 2014). The electrophysiological effects of this manipulation on Purkinje cells and their primary target, the cerebellar nuclei neurons, have also been extensively reported (Brown et al., 2020; van der Heijden et al., 2022; White et al., 2014). Owing to the severe and reproducible ataxic phenotype of the *L7^Cre^;Vgat^fx/fx^* mice, we were presented with an opportunity to explore sleep processes in the context of cerebellar ataxia. Furthermore, due to the highly restricted nature of our genetic manipulation that targets Purkinje cells, we could specifically test the interaction between cerebellar dysfunction and sleep.

In this study, *L7^Cre^;Vgat^fx/fx^* mice exhibited sleep impairments reminiscent of those reported in human patients with ataxia. Although the circadian activity of these mice remained intact, these mutants presented with altered sleep parameters and distinct electrocorticography (ECoG) patterns compared to those seen in controls. Specifically, they showed reduced wakefulness and REM sleep, increased NREM sleep, and prolonged latency to REM sleep – a finding aligned with prior research on human patients with ataxia (Sonni et al., 2014). Spectral analysis of ECoG signals indicated changes in power distribution across sleep-defining frequency bands in both frontal and parietal cortices, indicative of disrupted sleep homeostasis mechanisms. Our results highlight the potential role of Purkinje cell dysfunction in modulating wakefulness and the balance of sleep stages, which may contribute to sleep complications in disorders such as ataxia.

## RESULTS

### Purkinje cell silencing in *L7^Cre^;Vgat^fx/fx^* mice occurs throughout the cerebellum

**Fig. 1.**
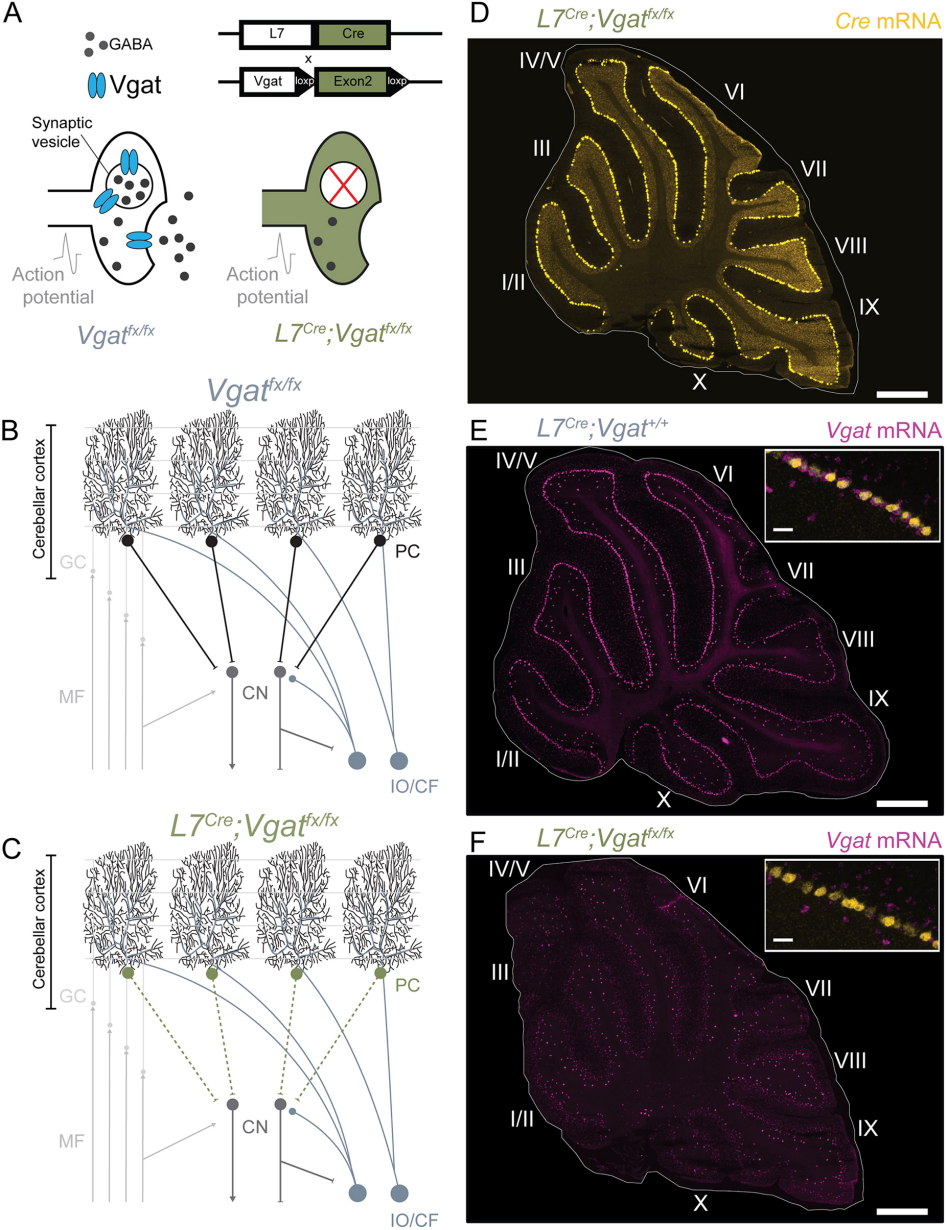
***L7^Cre^;Vgat^fx/fx^* mice display *Vgat* deletion throughout the cerebellar cortex.** (A) Using the *L7^Cre^* genetic driver line, exon 2 of *Vgat* was selectively removed from Cre-expressing cells. This resulted in deletion of VGAT expression with spatial sensitivity and subsequent silencing in the affected cells. (B) Schematic demonstrating a simplified cerebellar circuit in *Vgat^fx/fx^* control mice, in which all components of cerebellar signaling are intact and functional. CN, cerebellar nuclei; GC, granule cell; IO/CF, inferior olive/climbing fiber; MF, mossy fiber; PC, Purkinje cell. (C) Schematic demonstrating the result of *Vgat* deletion in *L7^Cre^;Vgat^fx/fx^* mutant mice. Mice displayed widespread silencing of Purkinje cell synaptic output. (D) Image acquired following *in situ* hybridization showing widespread *L7*-mediated *Cre* expression throughout the cerebellar cortex on adult mouse sagittal cerebellar tissue sections in an *L7^Cre^;Vgat^fx/fx^* mutant mouse. (E) Images were acquired as in D for *Vgat* expression in a control mouse carrying *L7^Cre^* but with no floxed copies of *Vgat*. The inset shows co-expression of *Cre* and *Vgat* in the cerebellar cortex. (F) Images were acquired as in E but for an *L7^Cre^;Vgat^fx/fx^* mutant mouse. The inset shows co-expression of *Cre* and *Vgat*, and lack of *Vgat* expression as expected. Roman numerals in D-F indicate the identity of the cerebellar lobules. Images are representative of two mice per genotype. Scale bars: 200 μm (D-F); 20 μm (insets in D-F).

In previous work, we demonstrated that genetically silencing Purkinje cell GABA neurotransmission can induce severe ataxic motor phenotypes in mice (Stay et al., 2019; White et al., 2014). The *L7^Cre^;Vgat^fx/fx^* mouse model uses the Cre-loxP system to drive the deletion of *Vgat* in neurons in which the *L7* promoter is active (Fig. 1A). In control (*Vgat^fx/fx^*) mice, Purkinje cells function normally with VGAT facilitating GABA loading into pre-synaptic vesicles. This ensures effective fast neurotransmission as GABA crosses the synaptic cleft, allowing action potential propagation. Anatomically, inhibitory efferents from the Purkinje cell layer in the cerebellar cortex synapse with the cerebellar nuclei neurons, the primary output cells of the cerebellum (Fig. 1B). In *L7^Cre^;Vgat^fx/fx^* mice, the loss of Purkinje cell *Vgat* results in the silencing of Purkinje cell-to-cerebellar nuclei neuron neurotransmission, as GABA cannot be loaded onto vesicles in the absence of VGAT (Fig. 1A,C). We validated that *L7*-mediated *Cre* expression in *L7^Cre^;Vgat^fx/fx^* mice was present throughout the cerebellar cortex (Fig. 1D), which resulted in a complete loss of *Vgat* expression in Purkinje cells (Fig. 1F). We have also confirmed the specificity of this deletion in previous works, using Purkinje cell-specific antibodies (White et al., 2014). Other cerebellar neuron types are present in their normal anatomical locations despite the manipulation (White et al., 2014). In *L7^Cre^;Vgat^+/+^* control mice, in which deletion of *Vgat* does not occur, *Vgat* expression was highly colocalized with *Cre* expression in the Purkinje cell layer of the cerebellar cortex (Fig. 1E). These findings confirm that the previously characterized ataxic phenotypes (impaired balance, lack of motor coordination and wide-based gait) are a result of silenced cerebellar Purkinje cells (Brown et al., 2020; White et al., 2014) and further support the use of this animal model to specifically probe the role of cerebellar Purkinje cells in regulating sleep.

### Circadian activity is unchanged in *L7^Cre^;Vgat^fx/fx^* mutant mice

**Fig. 2.**
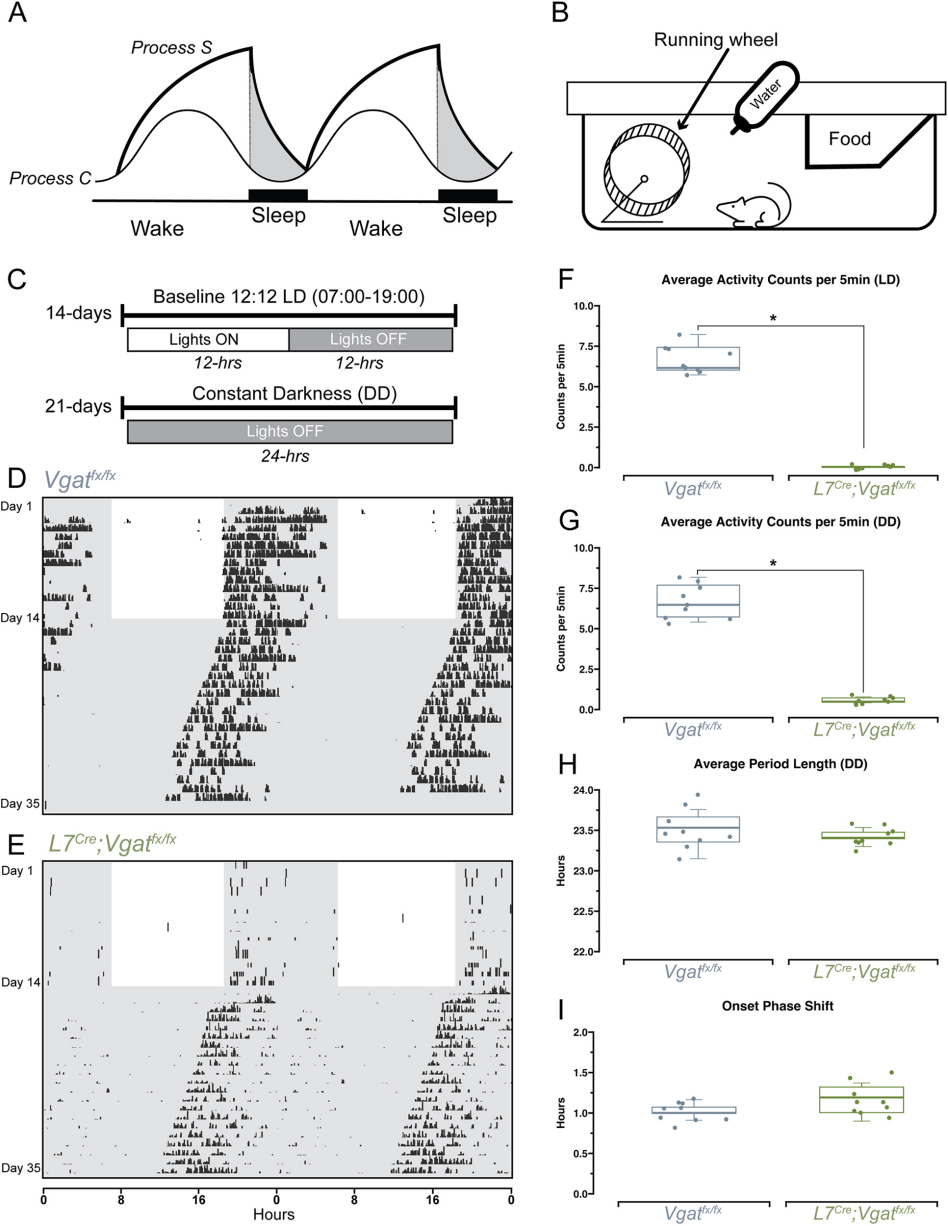
**Circadian wheel-running behavior is normal in *L7^Cre^;Vgat^fx/fx^* mutant mice.** (A) A schematic representation of the two-process model of sleep regulation. Process S denotes the homeostatic drive to sleep and process C denotes the circadian drive to sleep. (B) Schematic illustration of the home cage setup for wheel running. (C) Timeline of the wheel-running experiment. LD, light-dark; DD, dark-dark. (D,E) Representative double-plotted actograms for a control (D) and an *L7^Cre^;Vgat^fx/fx^* mutant (E) mouse. Rows represent days, and black ticks represent revolutions of the wheel, indicative of locomotor activity. Unshaded regions represent lights on, shaded regions represent lights off. (F,G) Quantification of average activity counts per 5 min during the LD phase (F) or the DD phase (G). (H) Quantification of average period length, only during the DD phase. (I) Quantification of onset phase shift, from day 14 to day 35 of recording. Boxes represent the interquartile range, whiskers show the maximum and minimum values, and the median is marked with a line. Points on F-I represent individual mice (*n*=9 per group). *P<0.05 (two-sample two-tailed unpaired *t*-test). All source data and specific *P*-values are available in Table S1.

Sleep regulation is thought to be governed by two processes: the homeostatic process (process S), which reflects the build-up of sleep pressure during wakefulness, and the circadian process (process C), which regulates the timing of sleep and wakefulness based on the 24-h biological clock (Borbély, 2022) (Fig. 2A). Wheel-running activity is used in rodents as a non-invasive proxy for measuring daily activity patterns (Eckel-Mahan and Sassone-Corsi, 2015), and previous work has used wheel running to assess circadian activity in other mouse models of mild ataxia (Mendoza et al., 2010) and dystonia (Salazar Leon and Sillitoe, 2023). As human patients with ataxia typically present with circadian deficits such as fatigue and excessive daytime sleepiness alongside changes in sleep quality (Sonni et al., 2014), we sought to determine whether circadian activity was similarly affected in *L7^Cre^;Vgat^fx/fx^* mutant mice. Mice were singly housed with *ad libitum* access to food, water and a running wheel within their home cage (Fig. 2B). Revolutions of the running wheels were automatically monitored for the entire duration of the recording period [14 days baseline, with a 12 h:12 h light-dark (LD) cycle, followed by 21 days under constant darkness (dark-dark or DD)], Fig. 2C). Data were collected and plotted as actograms, in which each row represents a day and black tick marks represent revolutions of the running wheel, indicative of locomotor activity, and are therefore interpreted as a period of wake. The data were double-plotted such that 48 h of activity is represented on a single line to better visualize the patterns of activity (Eckel-Mahan and Sassone-Corsi, 2015). We observed normal nocturnal behavior for *Vgat^fx/fx^* control mice for the 14-day baseline LD period, followed by free-running behavior in the DD period (Fig. 2D). Given their severe ataxic phenotype, we predicted that wheel-running behavior in *L7^Cre^;Vgat^fx/fx^* mice would be limited, and indeed found that mutant mice required significant time to begin running consistently (Fig. 2E). Interestingly, we did observe some activity that was qualitatively normal compared to that in controls, particularly during the DD period (Fig. 2E). As expected, average activity counts for *L7^Cre^;Vgat^fx/fx^* mutant mice were significantly lower during both LD and DD periods (Fig. 2F,G). Average period length (the time it takes for a circadian rhythm to complete a full cycle) during the DD condition was not significantly different between mutants and controls, suggesting that endogenous circadian activity remained intact (Fig. 2H). We also measured onset phase shift, which is the change in the timing of the onset of activity (the beginning of the active phase) in response to a manipulation. Here, the manipulation is the change from LD to DD conditions, and onset phase shift measures the gradual shift in the timing of activity onset from day 14 (DD start) until day 35 (experiment end). We found that onset phase shift was similar between *L7^Cre^;Vgat^fx/fx^* mutant mice and controls (Fig. 2I). These results suggest that circadian activity remains unchanged in *L7^Cre^;Vgat^fx/fx^* mutant mice, despite their cerebellar and motor dysfunction.

### Ataxic *L7^Cre^;Vgat^fx/fx^* mice display significantly disrupted stages of sleep

**Fig. 3.**
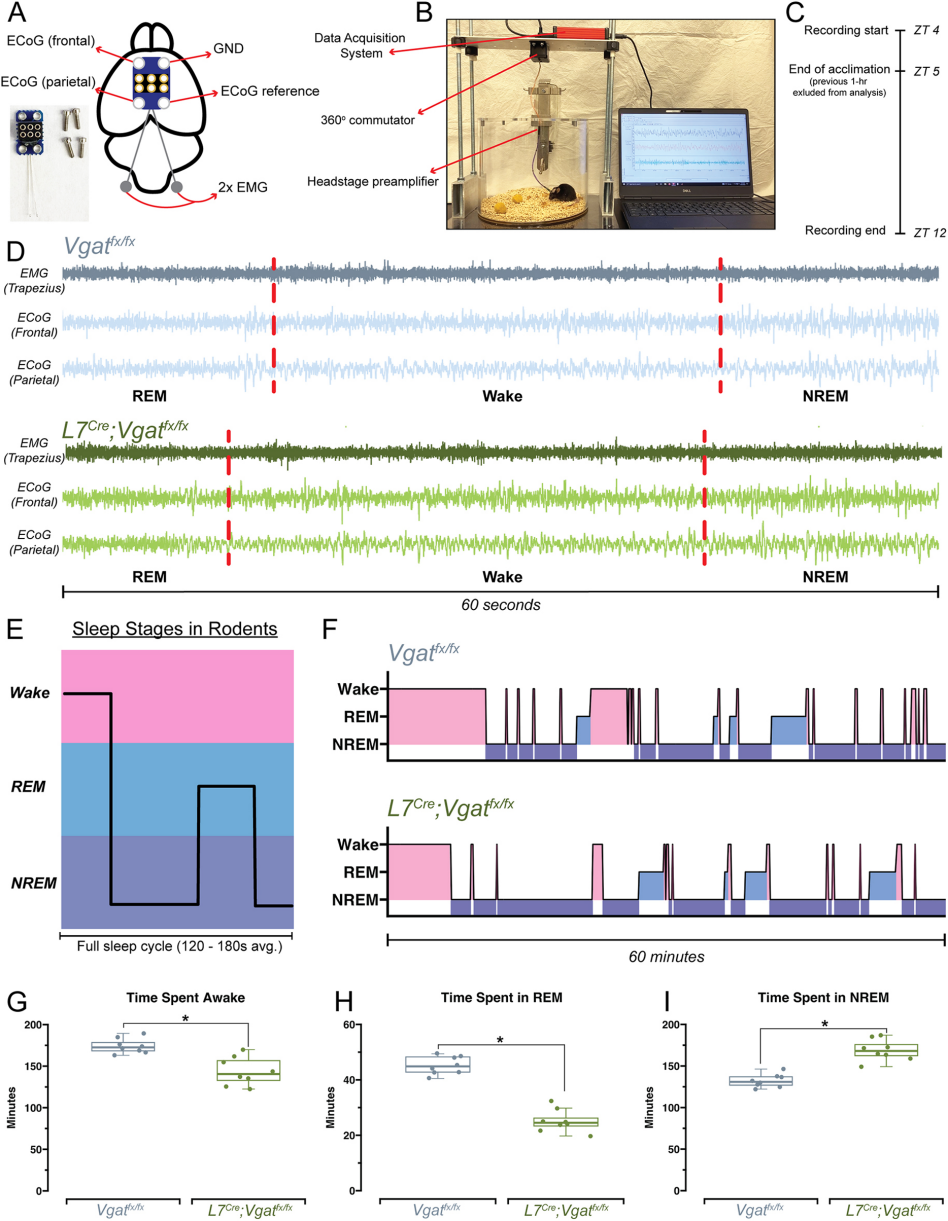
**Sleep patterns are disrupted in *L7^Cre^;Vgat^fx/fx^* mutant mice.** (A) Schematic illustration of a mouse brain, with electrocorticography (ECoG)/electromyography (EMG) headmount electrode placement. An image of the ECoG/EMG electrode and recording screws is also shown in the bottom left. GND, ground. (B) An image of the ECoG/EMG sleep-recording setup. (C) A schematic of the experimental timeline for recording sleep from each mouse. ZT, Zeitgeber time. (D) Raw waveforms of EMG (top trace) and ECoG (bottom traces) recorded from a control and a *L7^Cre^;Vgat^fx/fx^* mutant mouse. The example sample traces are 60 s in length. The sleep stages are noted at the bottom of each trace and differentiated by dashed red lines. REM, rapid eye movement; NREM, non-REM. (E) Schematic demonstrating sleep stages and their relative depth and temporal organization in mice. (F) Hypnograms for one representative *Vgat^fx/fx^* control mouse (top) and *L7^Cre^;Vgat^fx/fx^* mutant mouse (bottom). Both hypnograms refer to the same 1-h period, from 13:00 to 14:00. Periods of wake, REM sleep and NREM sleep are highlighted and correspond to the example schematic in E. (G-I) Quantification of total time spent awake (G), in REM sleep (H) and in NREM sleep (I). Boxes represent the interquartile range, whiskers show the maximum and minimum values, and the median is marked with a line. Points on G-I represent individual mice (*n*=8 per group). *P<0.05 (two-sample two-tailed unpaired *t*-test). All source data and specific *P*-values are available in Table S2.

The relationship between sleep and cerebellar ataxia is particularly relevant. Not only do patients with ataxia display disrupted sleep (Patterson et al., 2018; Shindo et al., 2019; Sonni et al., 2014), but ataxic symptom severity is also directly associated with sleep dysfunction. This combination subsequently has a direct impact on additional factors that affect quality of life, including fatigue and depression (Patterson et al., 2018; Sonni et al., 2014). Therefore, a major goal was to determine the sleep architecture of *L7^Cre^;Vgat^fx/fx^* mice. To do so, we implanted *L7^Cre^;Vgat^fx/fx^* mutants and *Vgat^fx/fx^* controls with platinum-iridium ECoG and electromyography (EMG) electrodes and recorded signals continuously during the light phase, when mice naturally sleep (Fig. 3A-C; Fig. S1). ECoG/EMG waveforms showed that *L7^Cre^;Vgat^fx/fx^* mice display the typical spectral activity, which defines the arousal states of wake, REM sleep and NREM sleep (Fig. 3D). We then assessed the total time spent in each arousal state for the duration of the recording period. We note that although typical sleep cycles in mice are shorter than in humans, they still follow the same general pattern of wake, followed by NREM sleep, followed by REM sleep (Fig. 3E). Hypnograms from 1 h of the recording period suggested that *L7^Cre^;Vgat^fx/fx^* mutants display disrupted sleep patterns relative to those of controls. Periods of wake were less frequent, whereas the lengths of individual bouts of NREM sleep were extended (Fig. 3F). Upon analyzing the proportions of time spent in each state for the entire recording period, we found that *L7^Cre^;Vgat^fx/fx^* mice spent significantly less time awake, less time in REM sleep and greater time in NREM sleep (Fig. 3G-I). These results suggest that the Purkinje cell-specific manipulation that alters neural activity in *L7^Cre^;Vgat^fx/fx^* mice is sufficient to drive impairments in sleep patterns, further supporting a role for the cerebellum in regulating sleep.

### Lack of Purkinje cell neurotransmission causes sleep pattern disruptions that can be defined by enhanced NREM sleep at the expense of wake and REM phases

**Fig. 4.**
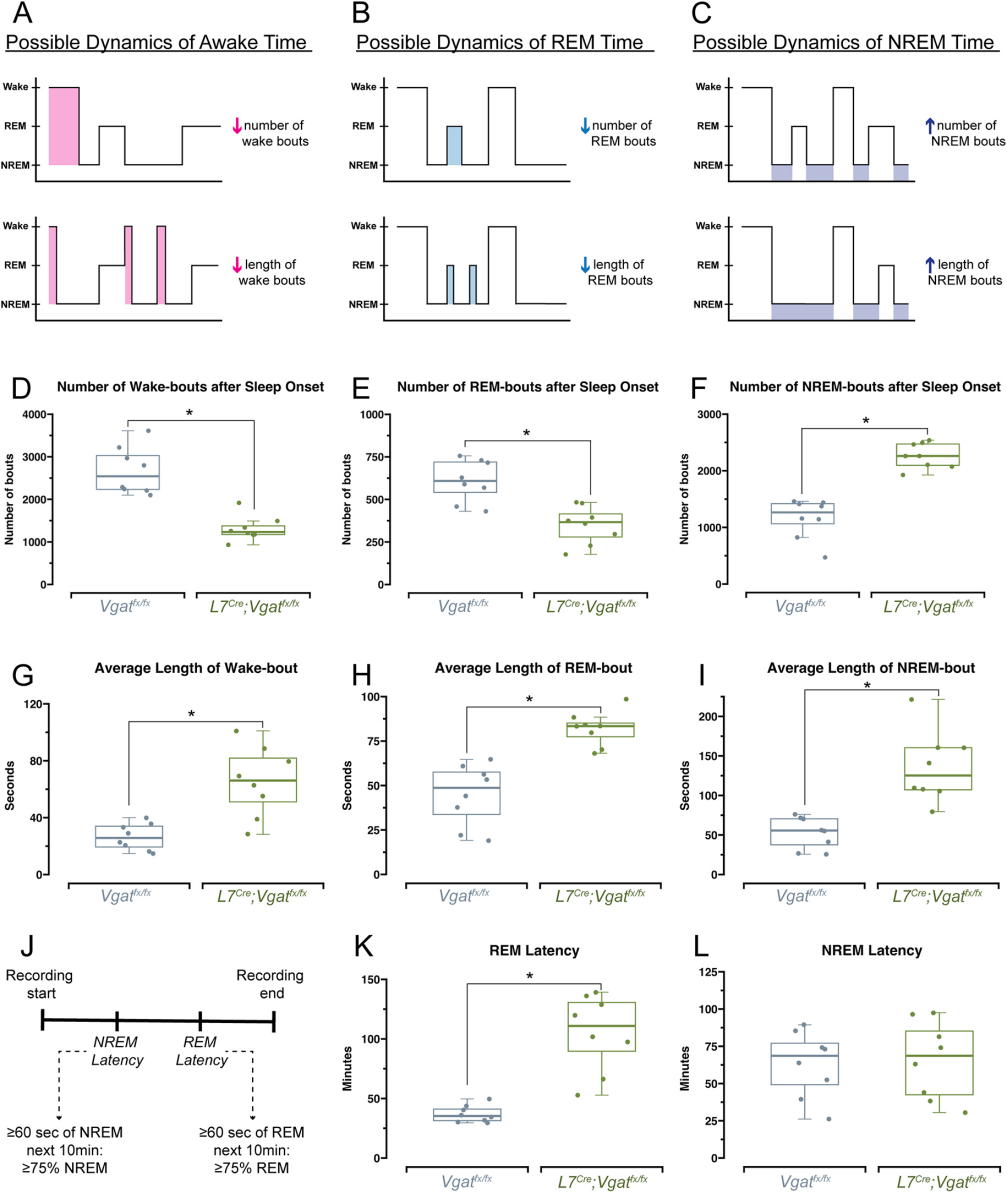
***L7^Cre^;Vgat^fx/fx^* mice have impairments in the quantity and length of sleep bouts.** (A-C) Schematic representation of hypnograms showing two hypothesized explanations for the changes in awake time (A), REM time (B) and NREM time (C), affecting the number of bouts or length of bouts. (D-F) Quantification of the number of awake bouts (D), REM bouts (E) and NREM bouts (F) after sleep onset. (G-I) Quantification of the average length of awake bouts (G), REM bouts (H) and NREM bouts (I). (J) Schematic showing how REM and NREM latency were calculated, as in Hunsley and Palmiter (2004). (K) Quantification of latency to REM sleep (K) and NREM sleep (L). Boxes in D-I,K,L represent the interquartile range, whiskers show the maximum and minimum values, and the median is marked with a line. Points represent individual mice (*n*=8 per group). *P<0.05 (Welch's two-sample two-tailed unpaired *t*-test). All source data and specific *P*-values are available in Table S3.

We observed that the Purkinje cell-initiated cerebellar ataxia in *L7^Cre^;Vgat^fx/fx^* mice was sufficient to disrupt sleep stages, with a particular impact on REM sleep. This is intriguingly similar to the patterns seen in human patients with ataxia, whose sleep disruptions disproportionally affect the timing and quality of REM sleep (Patterson et al., 2018; Pedroso et al., 2011; Sonni et al., 2014). Still, it was unclear how these patterns of disruption arose and whether changes in frequency or length of sleep stages (or both) were responsible for driving the observed sleep impairments (Fig. 4A-C). Therefore, to further understand the specific disruption of each stage, we assessed the total number of arousal-state bouts and the average length of each bout for wake, REM sleep and NREM sleep. All calculations were performed after the defined onset of sleep, which was determined according to previous work (Hunsley and Palmiter, 2004; Salazar Leon and Sillitoe, 2023). Consistent with our initial findings of sleep disruption, we found that the total numbers of wake bouts were significantly lower in *L7^Cre^;Vgat^fx/fx^* mice (Fig. 4D). Similarly, the total number of REM bouts was decreased, whereas the total number of NREM bouts was increased (Fig. 4E,F). Interestingly, we found that despite their overall decreased occurrence, the average length of wake bouts was greater for *L7^Cre^;Vgat^fx/fx^* mutant mice, suggesting the presence of a barrier to falling asleep or a reduced sleep pressure (Fig. 4G). We also found that the average length of both REM and NREM bouts was greater in *L7^Cre^;Vgat^fx/fx^* mice (Fig. 4H,I). Previous work in human patients suggests a deficit not only in the quantity of REM sleep, but also in the time to initially achieve REM sleep (Sonni et al., 2014) (REM latency). Given our results of decreased REM time, we hypothesized that a similar deficit for REM latency exists in the *L7^Cre^;Vgat^fx/fx^* mice. To this end, we calculated the latency to reach both REM and NREM sleep, to assess whether sleep disruption in *L7^Cre^;Vgat^fx/fx^* mice is primarily related to difficulties in falling asleep or staying asleep (or both) (Fig. 4J). Both the *L7^Cre^;Vgat^fx/fx^* mice and the *Vgat^fx/fx^* controls displayed similar NREM latency (Fig. 4L). However, the *L7^Cre^;Vgat^fx/fx^* mice had significantly elevated latency to REM sleep, by over 1 h (Fig. 4K). Together, these experiments define the specific sleep deficits in *L7^Cre^;Vgat^fx/fx^* mice as an overall increase in NREM time, due to an increase in number and duration of NREM bouts, at the expense of time spent in both wake and REM sleep. Although there is an increase in the duration of wake and REM bouts, this increase in duration cannot overcome the overall reduction in the number of wake and REM bouts, resulting in an overall reduction of time spent awake and in REM sleep. The deficits in REM sleep extend to the latency to achieve REM sleep, which is prolonged in *L7^Cre^;Vgat^fx/fx^* mice. These experiments further highlight the degree to which the quality, quantity and overall timing of wake and sleep phases are dependent on Purkinje cell GABA neurotransmission and support the occurrence of REM sleep deficits in ataxia.

### Sleep state impairments in *L7^Cre^;Vgat^fx/fx^* mice correspond to alterations in delta, theta, alpha, beta and gamma frequency bands across the frontal and parietal cortices

**Fig. 5.**
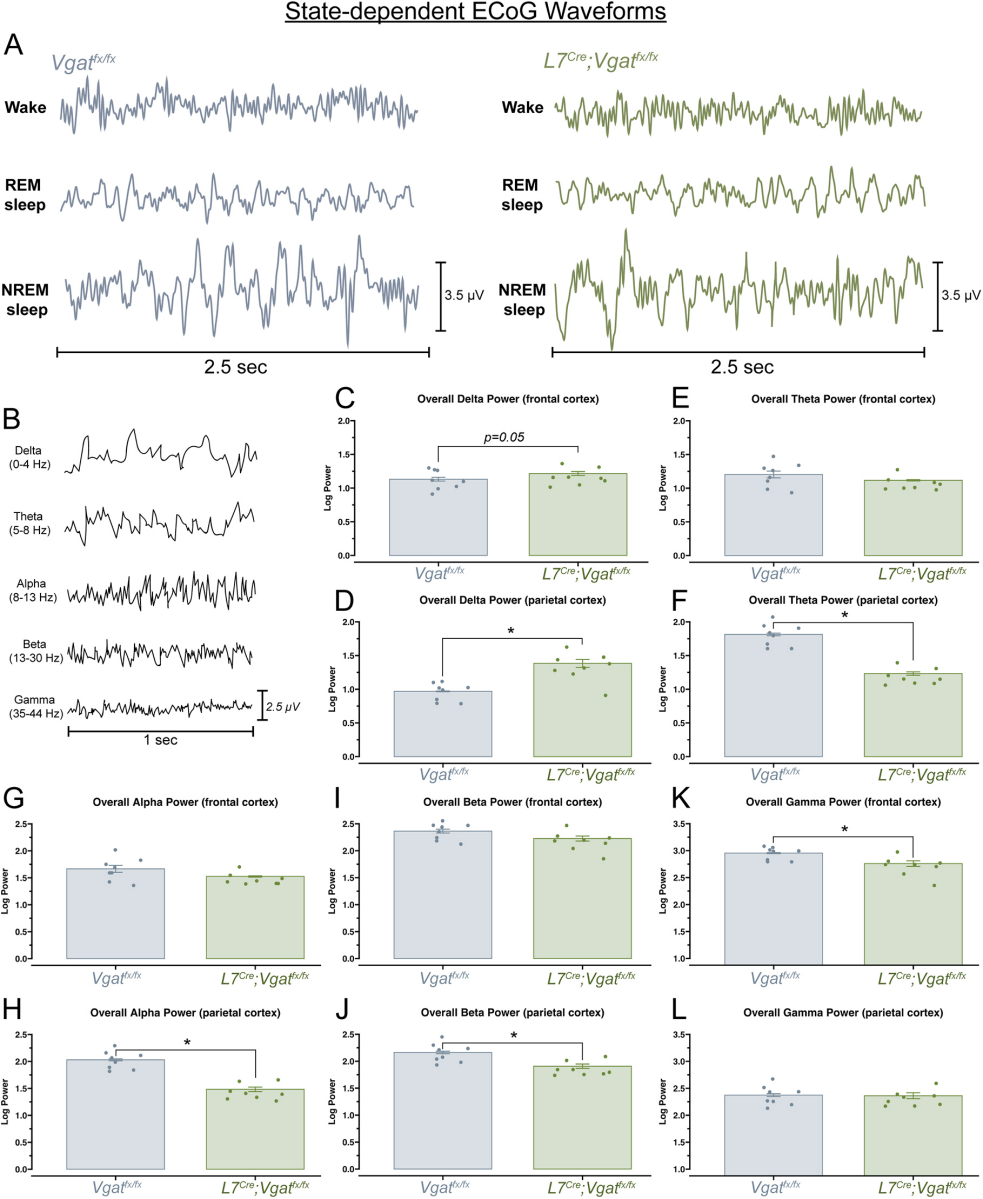
**Changes in delta, theta, alpha, beta and gamma frequency bands accompany sleep impairments in *L7^Cre^;Vgat^fx/fx^* mice.** (A) 2.5 s examples of raw ECoG waveforms of wake, REM sleep and NREM sleep from a *Vgat^fx/fx^* (control) and a *L7^Cre^;Vgat^fx/fx^* (mutant) mouse. (B) 1 s examples of raw ECoG waveforms from a *Vgat^fx/fx^* control mouse, for each frequency band of interest. (C,D) Quantification of overall delta power (0-4 Hz) over the frontal cortex (C) and the parietal cortex (D). (E,F) Quantification of overall theta power (5-8 Hz) over the frontal cortex (E) and the parietal cortex (F). (G,H) Quantification of overall alpha power (8-13 Hz) over the frontal cortex (G) and the parietal cortex (H). (I,J) Quantification of overall beta power (13-30 Hz) over the frontal cortex (I) and the parietal cortex (J). (K,L) Quantification of overall gamma power (35-44 Hz) over the frontal cortex (K) and the parietal cortex (L). Bars in C-L represent average (±s.e.m.) power across the entire recording period. Points represent individual mice (*n*=8 per group). *P<0.05 (Wilcoxon rank sum exact test). All source data and specific *P*-values are available in Table S4.

Wake, REM and NREM arousal states are defined by specific spectral frequency oscillations, which occur at frequency bands ranging from 0.5 to >100 Hz (Fig. 5A,B; Fig. S2). Transitions between sleep stages in mice can be described in part by changes in delta (0.5-4 Hz), theta (5-8 Hz) and alpha (8-13 Hz) frequency bands, which primarily correspond to NREM, REM and awake arousal states, respectively (Bjorness et al., 2018; Long et al., 2021). However, changes in higher-frequency bands, including beta (13-30 Hz) and gamma (35-44 Hz) frequency bands, can indicate not only disruptions in other neuronal processes such as associative memory consolidation or sensory processing (Posada-Quintero et al., 2019), but may also play a role in sleep-specific processes such as the maintenance of sleep homeostasis (Grønli et al., 2016). In this way, understanding the changes in spectral frequency oscillations can help to better frame the changes in sleep-wake dynamics, particularly as different frequency bands can be used to report overall changes in brain connectivity (Torres-Herraez et al., 2022). We therefore determined whether *L7^Cre^;Vgat^fx/fx^* mice displayed quantifiable differences in spectral frequency oscillations in the delta, theta, alpha, beta and gamma frequency bands across both recording electrodes (Fig. 5B). The same cortical (ECoG) electrodes used to record sleep states were used to detect changes in oscillation spectral power frequency throughout the recording period. The two independent ECoG electrodes were placed above the frontal and parietal cortices, and average spectral power from each region, for each frequency band of interest, was assessed. The *L7^Cre^;Vgat^fx/fx^* mice displayed significantly elevated delta power as measured over the parietal cortex, but not for the frontal cortex (*P*=0.05) (Fig. 5C,D). Theta (Fig. 5E,F), alpha (Fig. 5G,H) and beta (Fig. 5I,J) power were significantly decreased in the *L7^Cre^;Vgat^fx/fx^* mice, but only in the parietal cortex. In contrast, gamma power was decreased in *L7^Cre^;Vgat^fx/fx^* mice only in the frontal but not in the parietal cortex (Fig. 5K,L). These data demonstrate that *L7^Cre^;Vgat^fx/fx^* mutants and *Vgat^fx/fx^* controls have measurable differences in the spectral frequency bands relevant for sleep and that these differences are not homogenous throughout the cerebral cortex. Additionally, the dynamics of these changes in spectral activity concur with the directionality of overall changes in wake, REM and NREM time, further reinforcing that they are a direct result of our cerebellar manipulation.

## DISCUSSION

In this study, we used an *L7^Cre^;Vgat^fx/fx^* genetic manipulation that targets Purkinje cells to alter the dynamics of arousal states. Although this manipulation resulted in ataxia that reduced overall movement (Fig. 2F,G), it did not affect circadian activity (Fig. 2D,E,H,I). However, the proportion of arousal states and patterning of the phases of sleep were severely disrupted in these mutant mice lacking Purkinje cell neurotransmission (Figs 3F-I and 4D-L). This was further evidenced by alterations in ECoG frequency band powers in the frontal and parietal cortices (Fig. 5). With our previous work (Salazar Leon and Sillitoe, 2023), we show that Purkinje cells may promote normal sleep patterning and contribute to its disruption in the context of ataxia.

**Fig. 6.**
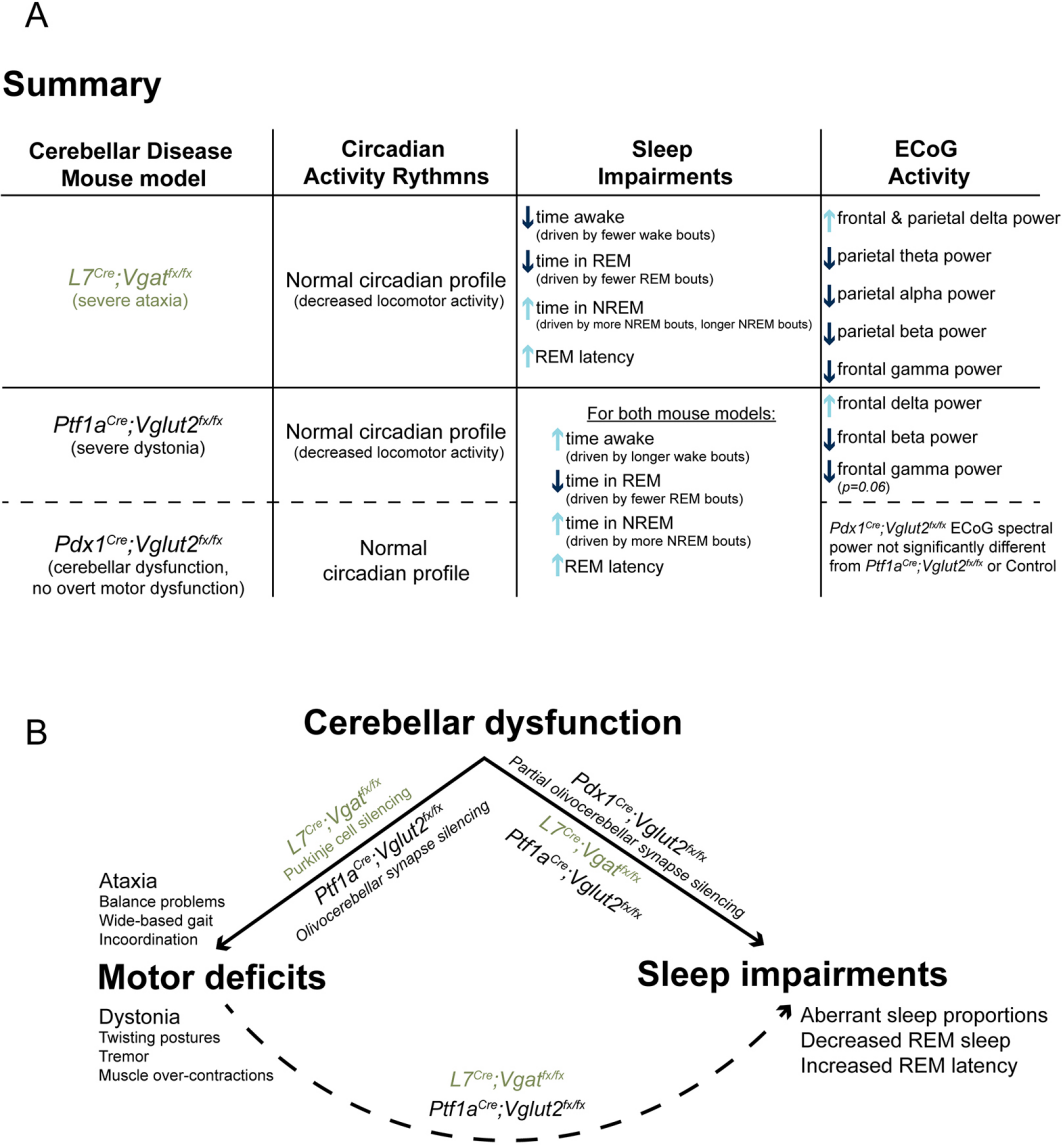
**Cerebellar dysfunction causes sleep impairments across multiple motor diseases.** (A) A summary of the main findings of this study, compared to the main findings from a complementary study in mouse models of dystonia from Salazar Leon and Sillitoe (2023). (B) A proposed model for the role of the cerebellum in regulating both motor functions and specific aspects of sleep homeostasis, using evidence from multiple animal models of motor disease.

Similar to in humans, mouse sleep stages follow specific temporal patterns (Fig. 3E) in which REM sleep (the lightest sleep stage) is typically preceded by NREM sleep (the deepest sleep stage) (Patel et al., 2022). Importantly, this means that sleep quality is determined not only by overall time spent sleeping, but also by the proportions of time spent in each state. Despite the emerging nature of research examining sleep dysfunction in patients with ataxia, existing studies suggest a prevalence of impaired sleep, with impacts on both sleep timing and quality (Huebra et al., 2019; Patterson et al., 2018; Pedroso et al., 2011; Shindo et al., 2019; Sonni et al., 2014). We observed similar changes in arousal state dynamics in *L7^Cre^;Vgat^fx/fx^* mice. We found that *L7^Cre^;Vgat^fx/fx^* mutant mice spent less time in REM sleep and, interestingly, also spent less time awake overall compared to the patterns seen in controls (Fig. 3G-I). Instead, they spent a significantly greater amount of time in NREM sleep. These shifted sleep proportions were driven by fewer wake and REM bouts, in favor of a greater number of NREM bouts (Figs 4D-I and 6A). Interestingly, the overall time spent in each arousal state was reflected by the number of bouts for wake, REM sleep and NREM sleep. However, the average duration of wake and REM bouts did not reflect the overall time spent in each state, as the average wake bout and REM bout were longer in the mutant mice (Fig. 4G,H), but not sufficient to offset the robust representation of bout number deficits. This contrasts with the patterns seen for NREM sleep, for which the average bout length was congruent with the overall time spent in this state (Fig. 4I). These results suggest that cerebellar dysfunction negatively drives sleep quality.

One possible explanation for the alteration of sleep patterning in *L7^Cre^;Vgat^fx/fx^* mice is that Purkinje cells have been found to have increased firing activity specifically during the transition from sleep to awake states (Zhang et al., 2020). As the *L7^Cre^;Vgat^fx/fx^* mouse model lacks Purkinje cell GABA neurotransmission, it is possible that the observed decrease in wake time is a result of our genetic manipulation via the removal of the ability of the Purkinje cells to properly signal the modulation of their activity during the transition out of sleep and into wakefulness. The downstream effect in the cerebellar nuclei is an increased regularity in firing pattern (Brown et al., 2020). This explanation is also parsimonious with our previous work in mouse models of dystonia that, in contrast, displayed an increase in total wake time (Salazar Leon and Sillitoe, 2023). In our dystonic mice, climbing fiber activity was genetically silenced. Previous work suggests that acute silencing of climbing fiber activity causes an increase in Purkinje cell firing rate (Cerminara and Rawson, 2004; Demer et al., 1985). Our constitutive silencing resulted in cerebellar nuclei activity with slower and more irregular firing patterns (White and Sillitoe, 2017). Therefore, it is possible that the abnormal cerebellar circuit activity in dystonia, driven by aberrant Purkinje cell activity, is more prone to triggering the awake state, whereas the complete silencing of Purkinje cells achieved in the *L7^Cre^;Vgat^fx/fx^* mouse model in this work achieves the opposite effect (Fig. 3G). The rationale behind this potential mechanism is strengthened by the established connections between the cerebellum and numerous cortical regions, which play roles not only in sleep regulation, but also in the management of specific sleep stages such as NREM and REM (Eban-Rothschild et al., 2016; Salazar Leon and Sillitoe, 2022; Van Dort et al., 2015). Additionally, there is a growing body of literature suggesting that the cerebellum regulates transitions between arousal states (Cunchillos and De Andrés, 1982; Zhang et al., 2020) and is involved in sleep features such as spindles (Canto et al., 2023 preprint; Xu et al., 2021) and behaviors such as sleep twitches (Canto et al., 2023 preprint; Dooley et al., 2021). In this way, our work here contributes to a growing model of cerebellar involvement in both motor and sleep dysfunction in the context of motor disorders, involving both cerebellar afferents and efferents (Fig. 6B) (Canto et al., 2017; Salazar Leon and Sillitoe, 2022).

Our results underscore the cerebellum as a possible mediator of REM-related sleep deficits in movement disorders. We demonstrated these deficits in our model by showing a decreased number of REM bouts (Fig. 4E), an increased duration of REM bouts (Fig. 4H) and an increased latency to REM sleep (Fig. 4K). The increased latency to REM sleep that we observed in the *L7^Cre^;Vgat^fx/fx^* mice (Fig. 4K) is particularly relevant, as it reflects observations not only from patients with ataxia (Shindo et al., 2019; Sonni et al., 2014), but from dystonia as well (Eichenseer et al., 2014; survey results from https://dystoniasurveys.org/), with the cerebellum playing a critical role in each disorder. It is possible to attribute this increased REM latency to motor dysfunction, as many REM-related sleep impairments are accompanied by involuntary motor function (Eichenseer et al., 2014; Shindo et al., 2019; Sonni et al., 2014; survey results from https://dystoniasurveys.org/) since the typical mechanisms of muscle atonia (a hallmark of REM sleep) are disrupted (Lydic, 2008). However, results from mouse models and humans have shown that sleep impairments in the context of motor disease can occur in the absence of motor symptoms (Antelmi et al., 2017; Salazar Leon and Sillitoe, 2023). In this case, dysfunction of the cerebellum and its circuit components may be to blame, although we acknowledge that motor dysfunction is reliably capable of causing disturbed sleep.

It is possible that, as REM and wake bouts are fewer in number, the increase in the bout lengths reflects some attempt of the brain to restore normal sleep homeostasis. Indeed, the ‘REM rebound effect’ is common in humans and rodents following sleep deprivation or after the presence of significant stressors, and involves the lengthening and increasing in the intensity of REM sleep, alongside decreased REM latency (Feriante and Singh, 2023). Although the existence of a similar ‘wake rebound’ is unknown, the increase in wake bout length may instead reflect the existence of some barrier to falling asleep or a decreased sleep pressure. It is known that patients with ataxia frequently report sleep-related involuntary motor behaviors such as restless leg syndrome, which typically interferes with the ability to go to sleep and stay asleep (Sonni et al., 2014). It is possible that a similar mechanism is responsible for the increased length of wake bouts in *L7^Cre^;Vgat^fx/fx^* mice. Alternatively, the greater number of NREM bouts may be akin to human naps, which reduce sleep pressure (Werth et al., 1996). Ultimately, however, these increases in wake and REM bout length are insufficient to overcome the deficits in the number of bouts, which primarily drive the observed global impairments in sleep proportions and timing.

It is known that, of the sleep centers in the brain that project to and from the cerebellum, many are directly involved in the regulation of REM sleep (Salazar Leon and Sillitoe, 2022). In particular, the locus coeruleus, which regulates NREM and REM intensity (Swift et al., 2018), sends dense projections to Purkinje cells and cerebellar nuclei neurons (Hoffer et al., 1973; Schwarz et al., 2015). Similarly, the pedunculopontine nucleus, another regulator of REM sleep (Romigi et al., 2008), has both afferent and efferent projections with the cerebellum, and with Purkinje cells in particular (Mori et al., 2016). Hence, the cerebellar dysfunction observed in *L7^Cre^;Vgat^fx/fx^* mice might exert a direct or indirect impact on REM latency. Although the direct versus indirect circuit pathways affecting sleep regulation were not mapped in this work, our results suggest that the Purkinje cell-specific manipulation in *L7^Cre^;Vgat^fx/fx^* mice is sufficient to disrupt sleep quality and has a particular impact on REM sleep.

It is intriguing that our data suggest deficits in the patterning of arousal states but not circadian activity. Evidence suggesting circadian dysfunction from mouse models of ataxia and human patients is mixed. Although results from animal models of mild ataxia suggest that overall circadian timekeeping ability remains intact (Mendoza et al., 2010), clinical studies suggest that human patients with both hereditary spinocerebellar ataxia and idiopathic cerebellar ataxia exhibit distinct abnormalities in their circadian rhythms, including fatigue and daytime sleepiness (Sonni et al., 2014). Our results show that *L7^Cre^;Vgat^fx/fx^* mice display normal circadian timing of behavior, suggesting that the Purkinje cell-specific manipulation is not sufficient to impact overall circadian behavior (Fig. 2F-I). Alternatively, it is also possible that the use of wheel running to assay circadian behavior in ataxic mice is not sufficiently sensitive to detect the more subtle circadian deficits (as overall *L7^Cre^;Vgat^fx/fx^* activity is lower due to the motor phenotype) and the use of home-cage monitoring systems that use infrared break-beam detection could be beneficial for future work. It is simultaneously possible that, although our wheel-running measures of circadian activity did not reveal impaired circadian timekeeping ability, the decrease in awake time that we observed (Fig. 3G) reflects the reported increases in fatigue and sleepiness in human patients with ataxia.

Although there are many projections between the cerebellum and key sleep centers of the brain, including the locus coeruleus (Moises et al., 1981), pedunculopontine nucleus (Mori et al., 2016) and the hypothalamus (Dietrichs and Haines, 1989), there are no known direct projections between the cerebellum and suprachiasmatic nucleus (Salazar Leon and Sillitoe, 2022; Van Dort et al., 2015). Therefore, in accordance with results from animal models of mild ataxia (Mendoza et al., 2010), our data suggest that the ability of the cerebellum to directly regulate circadian activity rhythms is limited. If this is the case, the fatigue and sleepiness observed in human patients with ataxia may be due to a lack of quality sleep, peripheral mechanisms of fatigue, or even the impact of other comorbid mood disorders such as depression, rather than a result of impaired circadian timekeeping ability.

Our ECoG spectral activity analysis offers insights into the potential mechanisms underlying sleep deficits. Although linking sleep disruptions to specific frequency band changes is challenging – given that power fluctuations across bands correlate with various disease states, including sleep disorders (Bjorness et al., 2018; Long et al., 2021) – we can interpret spectral frequency power shifts as indicative of disrupted sleep homeostasis in our ataxia mouse model. Such insight is crucial as it helps in determining the broader impacts of cerebellar dysfunctions on brain activities, particularly in the context of sleep. For instance, we observed an increase in delta power for *L7^Cre^;Vgat^fx/fx^* mice in the parietal cortex (Fig. 5D). This finding is reminiscent of independent work in humans showing that higher delta power is associated with sleep impairments, such as in instances of obstructive sleep apnea (Liu et al., 2021). This is of particular interest as obstructive sleep apnea or general instances of sleep-disordered breathing is not only prevalent in patients with ataxia, but also thought to be regulated in part by the cerebellum (Corben et al., 2013; Kapoor and Greenough, 2015; Liu et al., 2020). We also observed a decrease in parietal beta power (Fig. 5J), which is similarly associated with obstructive sleep apnea in human patients. Beta power is also an indicator of alert wakefulness, which can be elevated in patients with primary insomnia (Liu et al., 2021). As *L7^Cre^;Vgat^fx/fx^* mice appeared to spend less time awake, the observed decrease in beta power may also be an indication of wake time reduction. Interestingly, bursts of beta activity can also occur during REM sleep (Steriade, 2009). As total REM time is lower in the mutant mice, this may also explain the observed decrease in beta power. Reductions in parietal theta power were also observed (Fig. 5F), which may reflect the overall reduction in REM sleep in *L7^Cre^;Vgat^fx/fx^* mice, as it is known that theta waves are predominant during REM sleep (Merica and Blois, 1997) and often are used to characterize REM sleep (Patel et al., 2022). Additionally, we observed a decrease in parietal alpha power (Fig. 5H). It is known that a decrease in alpha power is associated with feelings of sleepiness in healthy adults (Strijkstra et al., 2003). The observed decreases in frontal gamma power (Fig. 5K) also have direct associations to impairments in sleep. Gamma power can reflect working memory and attention, which may be expected to decrease in *L7^Cre^;Vgat^fx/fx^* mice, which display decreased awake time (Goddard et al., 2012). Spontaneous gamma activity also occurs during REM sleep (Steriade, 2009); the decreased gamma power may then reflect the observed decrease in REM duration. We note that region-specific variations in electrographic activity are prevalent in the literature, with differential sleep-related spectral power typically observed between frontal and posterior regions of the cortex in non-disease states (Soltani et al., 2019). In contrast, global shifts in spectral power are often associated with specific disease states, such as epilepsy (Yang et al., 2012). Therefore, our observation of spectral power differences between parietal and frontal cortices across various frequency bands aligns with established patterns in the field.

It is intriguing to consider how the ECoG activity may be altered within specific arousal states in our ataxic mice. The frequency bands analyzed in this work are not restricted to specific arousal states and, therefore, measuring band power by arousal state could provide further insights into the ECoG phenotype of our ataxic animals. Interestingly, our results are reminiscent of findings in mice in response to high doses of ethanol. Acute administration of ethanol results in ethanol-induced ataxia that, similar to the ataxia of the *L7^Cre^;Vgat^fx/fx^* mice, is an ataxia not caused by neurodegeneration thought to involve the cerebellar circuit (Dar, 2015). High doses of ethanol have been found to increase time spent in NREM sleep, reduce time spent awake and in REM sleep, and increase REM latency (Abrahao et al., 2020; Fang et al., 2017), which is similar to the proportion and timing of arousal states we found in *L7^Cre^;Vgat^fx/fx^* mice (Figs 3 and 4). There are also comparable changes in overall frequency band power, with delta power increased and higher-frequency bands suppressed (Fig. 5). In the ethanol model, delta power has been found to be significantly increased during NREM sleep and suppressed during wake and REM sleep. Higher-frequency bands have been found to be suppressed in all arousal states (Abrahao et al., 2020). It is therefore conceivable that a similar distribution of band power occurs in *L7^Cre^;Vgat^fx/fx^* mice. It is important to note that the degenerative ataxias may have different underlying circuit alterations from those in the non-degenerative ataxias (van der Heijden et al., 2023), potentially resulting in different ECoG profiles and sleep disturbances. Interestingly, this has been investigated in a spinocerebellar ataxia 3 (SCA3) mouse model, in which different distributions of bout duration and number were found compared to what we report in *L7^Cre^;Vgat^fx/fx^* mice and what has been reported in ethanol-induced mice (Tsimpanouli et al., 2022). This is perhaps unsurprising due to the breadth of degeneration that occurs in SCA3, which involves not only the cerebellum, but also the brainstem, basal ganglia and cerebral cortical regions. These include key areas involved in sleep, such as the locus coeruleus (Burright et al., 1997). In contrast, our *L7^Cre^;Vgat^fx/fx^* model allows investigation of not only brain region-specific, but also cell type-specific contributions to sleep disturbances.

In conclusion, by exploiting the genetic precision of the *L7^Cre^;Vgat^fx/fx^* mouse circuit model of ataxia, we tested whether sleep regulation depends on cerebellar Purkinje cells. Our data support the possibility of a critical role for the cerebellum in sleep regulation, which is reflected in the patterns of sleep disruption that are observed in human movement disorders. These findings not only expand our understanding of the involvement of the cerebellum in nonmotor complications in motor diseases, but also suggest that the cerebellar circuitry drives similar sleep deficits across different motor disorders (Fig. 6B). This knowledge points to a potential broader network dysfunction in motor disorders, with the cerebellum and its circuits poised at the nexus of various disease symptoms.

## MATERIALS AND METHODS

### Animals

All mice used in this study were housed in a level 3, American Association for Laboratory Animal Science (AALAS)-certified facility that operates on a 14-h light cycle. Husbandry, housing, euthanasia and experimental guidelines that involved mice were reviewed and approved by the Institutional Animal Care and Use Committee of Baylor College of Medicine (protocol number: BCM AN-5996). We purchased *L7^Cre^* (L7Cre-2, #004146) (Lewis et al., 2004) and *Vgat*-floxed (*Vgat^flox^*, #012897) (Tong et al., 2008) mice from The Jackson Laboratory (Bar Harbor, ME, USA) and then maintained them in our colony using a standard breeding scheme. The conditional knockout mice that resulted in ataxia were generated by crossing *L7^Cre^;Vgat^fx/fx^* heterozygote mice with homozygote *Vgat^fx/fx^* mice. *L7^Cre^;Vgat^fx/fx^* mice were considered experimental animals. A full description of the genotyping details (e.g. primer sequences and the use of a standard polymerase chain reaction) has been provided previously in White et al. (2014). All littermates lacking *Cre* upon genotyping were considered control mice. Ear punches were collected before weaning and used for genotyping and identification of the different alleles. For all experiments, we bred mice using standard timed pregnancies, noon on the day a vaginal plug was detected was considered embryonic day (E) 0.5, and postnatal day (P) 0 was defined as the day of birth. Mice of both sexes were used in all experiments.

### Tissue preparation and processing for *in situ* hybridization

mRNA *in situ* hybridization (ISH) was performed on freshly frozen 25 µm-thick sagittal brain sections cut through the cerebellum. Sections were cut using a cryostat (Leica, CM3050) and collected on Superfrost Plus microscope slides (Thermo Fisher Scientific). We generated digoxigenin (DIG)-labeled mRNA antisense probes against *Cre* or *Vgat* using reverse-transcribed mouse cDNA as a template and an RNA DIG labeling kit from Roche (Sigma-Aldrich). Primer and probe sequences for the *Cre* and *Vgat* probes are available on the Allen Brain Atlas website (http://www.brain-map.org). Sectioning and ISH were performed by the RNA *In Situ* Hybridization Core at Baylor College of Medicine. The core used an ISH protocol and an automated robotic liquid handling platform for the procedure as previously described (Yaylaoglu et al., 2005).

### Wheel-running behavior

Recordings were maintained in a ventilated, temperature-controlled and light-tight room under either a 12 h:12 h light-dark (LD) cycle or dark-dark (DD) conditions. Mice were singly housed in wheel-running cages and allowed to entrain to the LD cycle for 2 weeks, before being released into DD conditions for 21 days to assess endogenous circadian timekeeping ability. We assessed period length, activity onset, phase shift onset and average number of wheel revolutions per 5 min using ClockLab Analysis (Actimetrics). All measures were calculated automatically by the Clocklab Analysis software.

### Surgical procedure for ECoG/EMG sleep recordings

Prior to surgery, mice were given preemptive analgesics (extended-release buprenorphine, 1 mg/kg subcutaneous injection, and meloxicam, 5 mg/kg subcutaneous injection) with continued application as part of the standard 3 day post-operative procedure. Mice were anesthetized with isoflurane and placed into a stereotaxic device, which continued to deliver isoflurane throughout surgery. Each mouse with implanted with a prefabricated ECoG/EMG headmount (Pinnacle Technology, Lawrence KS, #8201) with 0.10″ EEG screws to secure headmounts to the skull (#8209, Pinnacle Technology, Lawrence, KS, USA). To do this, fur was removed with depilatory cream (Nair) and the surgical site was sterilized with alternating applications of alcohol and betadine scrub solution. Then a midline incision was made, and the skull was exposed. The headmount was affixed to the skull using cyanoacrylate glue to hold it in place while pilot holes for screws were made and screws were inserted. Screws were placed bilaterally over the parietal cortex and frontal cortex. A small amount of silver epoxy (#8226, Pinnacle Technology) was applied to the screw-headmount connection. Platinum-iridium EMG wires on the prefabricated headmount were placed under the skin of the neck, resting directly on the trapezius muscles. The headmount was permanently affixed to the skull using ‘Cold-Cure’ dental cement (#525000 and #526000, A-M Systems). Mice were allowed to recover for 3-4 days before being fitted with a preamplifier (#8202, Pinnacle Technology) and tethered to the recording device (#8204 and #8206-HR, Pinnacle Technology).

### ECoG/EMG sleep recordings

Mice were recorded in light- and temperature-controlled rooms for 8 h at the same time of day for every mouse. The first hour of recording was considered the acclimation period and was therefore excluded from final analysis. Food and water were available *ad libitum* throughout the recording day. Mice were singly housed in clear acrylic cages (#8228, Pinnacle Technology). Preamplifiers were connected to a 360° commutator allowing for unrestricted movement (#8204, Pinnacle Technology). All data were collected by the Data Acquisition and Conditioning System (#8206-HR, Pinnacle Technology) which was specifically tuned for detecting sleep. Data were captured using Sirenia Acquisition software (Pinnacle Technology).

### Sleep scoring and analysis of sleep data

Sleep was automatically scored offline via the ‘Sleep Phase Identification with Neural networks for Domain-invariant LEearning’ (SPINDLE) method according to the built-in functionalities of the neural network (Miladinović et al., 2019). As part of preliminary validation of the automated process, initial recordings were manually scored for validation as in previous works (Sanchez et al., 2019). SPINDLE relies in part on frequency domain changes to score sleep stages. The SPINDLE algorithm extends beyond band power analysis; it incorporates multiple spectral features, assesses both ECoG and EMG activity together, and uses specific preprocessing techniques to enhance spectral pattern consistency within each recording. Furthermore, SPINDLE has been trained and validated across diverse rodent models, reinforcing its reliability amidst abnormal brain rhythms (Miladinović et al., 2019). For spectral frequency analysis of ECoG and EMG activity, raw files were also pre-processed in MATLAB (MathWorks) using the free toolkit EEGLAB (Delorme and Makeig, 2004). The definition of arousal states (wake, REM sleep and NREM sleep) was determined using the SPINDLE algorithm by classifying ECoG and EMG data. Data were binned in 10-s intervals and power spectra of both ECoG and EMG signals was performed (Welch's method, with a 50% overlapping window). In calculating the ECoG band power values, the average of the values measured from the frontal and parietal cortexes was used. Key features were extracted as follows: delta power, defined as ECoG power in the 0.5-4 Hz frequency band (a feature of NREM sleep); theta power, defined as ECoG power in the 5-8 Hz frequency band (a feature of REM sleep); alpha power, defined as the ECoG power in the 8-13 Hz frequency band (a feature of wake); and the root mean square (RMS) of the EMG signal. The SPINDLE algorithm first classifies bins as either wake or sleep depending on whether the ECoG power or EMG RMS exceeds defined thresholds. For the ECoG power, SPINDLE determines the alpha power/(theta power/delta power) value for each bin during the recording and determines whether it exceeds the average for the animal over the duration of the recording plus one standard deviation. If so, the bin is considered ‘wake’. Additionally, SPINDLE determines whether the EMG RMS exceeds a threshold of 0.4 standard deviations above the mean. If this threshold is crossed, the bin is considered as ‘wake’. This combination of ECoG and EMG is beneficial for correctly classifying the time during which the mouse is relaxed and awake. If neither the ECoG nor EMG thresholds are met, the bin is labeled as ‘sleep’. ECoG power is used to further differentiate sleep bins between REM sleep and NREM sleep. A bin is classified as ‘REM sleep’ if the theta-to-delta power ratio exceeds the average for the animal plus one standard deviation. All other sleep bins are identified as ‘NREM sleep’. This method using the SPINDLE algorithm is based on previous approaches for manual sleep scoring and has been validated in independent studies (Hsu et al., 2017; Sanchez et al., 2019).

### Data analysis and statistics

Data are presented as mean±s.e.m. and were analyzed with a two-sample two-tailed unpaired *t*-test (normally distributed data with equal variance), Welch’s two-sample two-tailed unpaired *t*-test (normally distributed data with unequal variance) or Wilcoxon rank sum exact test (non-normally distributed data with unequal variance). For all statistical tests, *P*<0.05 was considered as statistically significant. All statistical analyses were performed using R v4.1.2.

## Supplementary Material

10.1242/dmm.050379_sup1 Supplementary information

Table S1. Source data and specific *P*-values for Fig. 2

Table S2. Source data and specific *P*-values for Fig. 3

Table S3. Source data and specific *P*-values for Fig. 4

Table S4. Source data and specific *P*-values for Fig. 5
