# Supplementary material for: Purkinje cell dysfunction causes disrupted sleep in ataxic mice
Source: Dis Model Mech. 2024 Jun 12;17(6):dmm050379. doi: 10.1242/dmm.050379 (PMC11190574; doi:10.1242/dmm.050379)
Supplement: Supplementary information [file dmm-17-050379-s1.pdf]

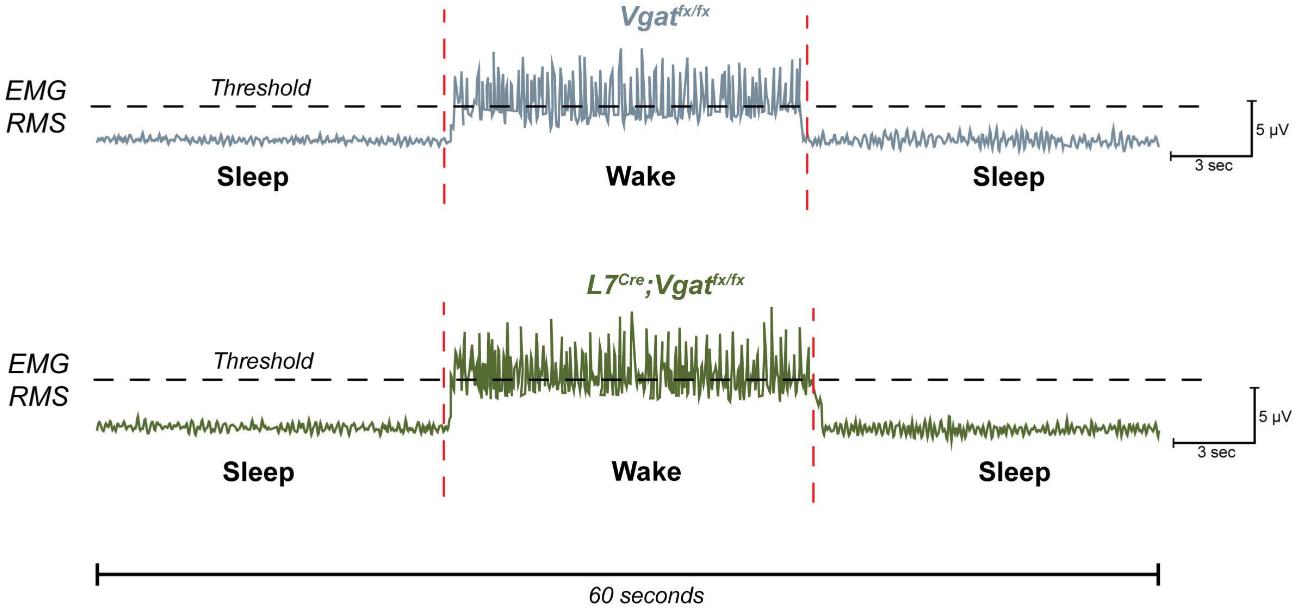

Fig. S1. EMG RMS is used to define sleep versus wake states. Waveforms showing RMS (magnitude) of EMG signals for representative *Vgat*$^{fx/fx}$ (top, gray) and *L7*^{Cre}*;Vgat*$^{fx/fx}$ (bottom, green) mice. Defined arousal state is noted in bold at the bottom of each waveform. Vertical dashed red lines indicate different arousal states. Horizontal dashed black lines indicate the EMG RMS threshold for classifying “Wake”.

Disease Models & Mechanisms • Supplementary information

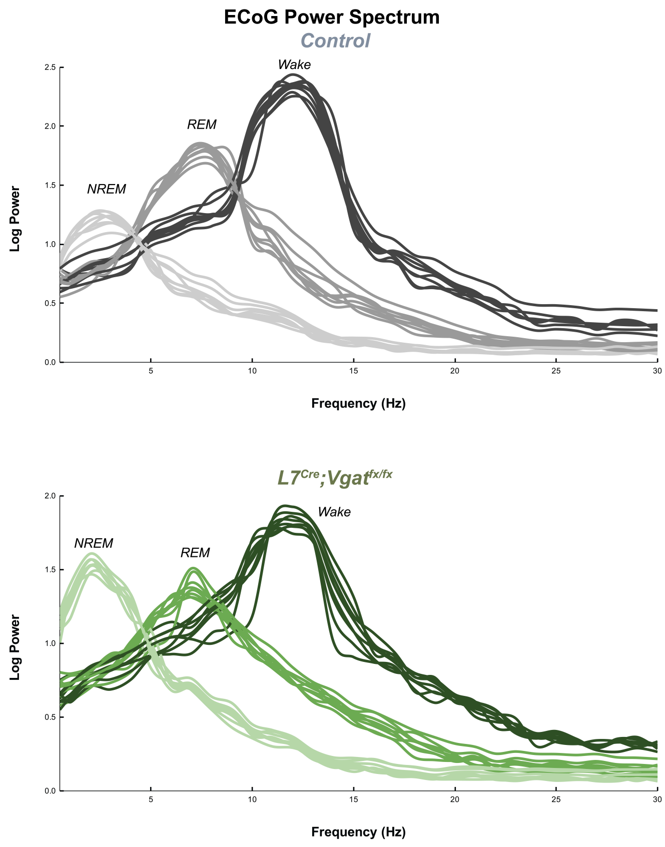

Fig. S2. Illustration of the power spectrums of ECoG signals in all 3 brain states. ECoG power spectrums averaged between frontal and parietal recordings for *Vgat*$^{fx/fx}$ (top, gray) and *L7*^{Cre};*Vgat*$^{fx/fx}$ (bottom, green) mice. N = 8 mice per group, one line per mouse.

Disease Models & Mechanisms • Supplementary information

Table S1. Source data and specific *P*-values for Fig. 2

Available for download at

https://journals.biologists.com/dmm/article-lookup/doi/10.1242/dmm.050379#supplementary-data

Table S2. Source data and specific *P*-values for Fig. 3

Available for download at

https://journals.biologists.com/dmm/article-lookup/doi/10.1242/dmm.050379#supplementary-data

Table S3. Source data and specific *P*-values for Fig. 4

Available for download at

https://journals.biologists.com/dmm/article-lookup/doi/10.1242/dmm.050379#supplementary-data

Table S4. Source data and specific *P*-values for Fig. 5

Available for download at

https://journals.biologists.com/dmm/article-lookup/doi/10.1242/dmm.050379#supplementary-data
